# Sex-based differences in anti-predator response of crickets to chemical cues of a mammalian predator

**DOI:** 10.7717/peerj.4923

**Published:** 2018-06-11

**Authors:** Brian P. Tanis, Bradley Bott, Brian J. Gaston

**Affiliations:** 1 Department of Integrative Biology, Oregon State University, Corvallis, OR, United States of America; 2 Department of Biological Sciences, Fort Hays State University, Hays, KS, United States of America; 3 PRA Health Sciences, Lenexa, KS, United States of America

**Keywords:** Anti-predatory behavior, Foraging, Chemical cues, Boldness, Predation risk, Sexual dimorphic, Sex ratio, Ecology of fear, Behavior, Shrew

## Abstract

Anti-predator behaviors like vigilance or hiding come at the expense of other fitness increasing behaviors such as foraging. To compensate for this trade-off, prey assess predation risk and modify the frequency of anti-predator behaviors according to the likelihood of the threat. In this study, we tested the ability of house crickets (*Acheta domesticus*) to indirectly assess predation risk via odors from a mammalian predator, Elliot’s short-tailed shrew (*Blarina hylophaga*). As natural differences in encounter rates and predation risk differs between sexes, we tested if male and female crickets perceive similar rates of predation risk from the presence of shrew odor measured via anti-predator behavioral response. Crickets were placed in enclosed, cardboard-lined chambers either treated with shrew odor or control, along with a food source. Time until foraging was measured for each individual and compared across treatment and sex. We found that in the presence of shrew odor, female crickets delayed foraging while males showed no response. These results suggest adult crickets can use chemical cues to detect mammalian predators. Furthermore, we demonstrate that female crickets associate greater predation risk from shrew predators than do male crickets, which are more stationary yet acoustically conspicuous. As predation risk potentially differs drastically for each sex, changes to the operational sex ratios of wild cricket populations could be influenced by the identity of the predator community.

## Introduction

Anti-predator behaviors (e.g., vigilance and hiding) are frequently associated with direct high fitness tradeoffs, including reduced time foraging or searching for mates (Brown, 1999; Lind & Cresswell, 2005). While costs of anti-predatory behaviors will always be less than mortality, lost opportunities during predator avoidance are significant enough that it benefits species to occasionally reduce or forego anti-predatory behaviors, also referred to as boldness (Main, Weckerly & Blech, 1996). Theoretical and empirical studies have shown that measuring differences in the frequency and duration of anti-predator behaviors correlates with the frequency of predation risk experienced (Lind & Cresswell, 2005; Preisser, Bolnick & Benard, 2005; Abbey-Lee, Mathot & Dingemanse, 2016).

One challenge in reconstructing the landscape of predation risk for a species is that this risk will frequently scale with variation in phenotypic traits (Werner & Peacor, 2003). Although applying to any phenotypic continuum, this pattern is exacerbated among species with pronounced sexual dimorphism (Croft et al., 2006). Sexually dimorphic traits such as coloration, body size, and ornamentation can alter the ability of predators to find, capture, and subdue prey (Brown, 1999; Croft et al., 2006). Physical attributes are not the only sexually dimorphic traits that impact predation risk; differences in behaviors will also impact the odds of predator-induced mortality. For instance, brooding behavior, specialized foraging needs, and conspicuous displays can also create unequal rates of predation between sexes (Wolfe et al., 2007).

Crickets (Orthoptera: Gryllidae) are frequently used as a model system for testing anti-predator behavior and provide a well-studied example of how sexually dimorphic behaviors can drive unequal rates of predation risk and boldness. As male crickets chirp to attract mates, they also signal acoustically-hunting parasitoids and predators. Silent females are inconspicuous to acoustically-hunting parasitoids and therefore tend to be significantly bolder than males in regions of high parasitoid density (Hedrick & Kortet, 2006). These findings have prompted insight into community ecology following novel dispersals and invasion biology (Trompeter & Langkilde, 2011; Cote et al., 2013), differentiation into adaptive behavioral suits (Wilson et al., 2010; Niemelä, DiRienzo & Hedrick, 2012; Hedrick & Kortet, 2012), and predator–prey evolutionary arms races (Zuk, Rotenberry & Tinghitella, 2006; Beckers & Wagner, 2012). However, not all cricket predators rely on acoustic signals to locate prey. Many predators, especially opportunistic generalists, rely on chance encounters while foraging (Iwasa, Higashi & Yamamura, 1981; Lucas, 1983; Bastille-Rousseau et al., 2011). Females, which must actively seek out stationary signaling males to mate, have an increased likelihood of encountering an opportunistic, mobile or sit-and-wait predator than males would. Therefore, female crickets are expected to have higher predation risk and reduced levels of boldness for non-acoustic hunters (Hedrick & Dill, 1993; Csada & Neudorf, 1995). Females, which are the more selective sex in crickets, also use a variety of other sensory cues for mate choice, including visual, chemical, and tactile (Otte & Cade, 1976; Hedrick & Dill, 1993; Adamo & Hoy, 1994; Nelson & Nolen, 1997; Gray, 1999; Thomas, 2011). In this study we set out to test the hypothesis that female crickets perceive a greater risk of predation from opportunistic mobile hunters compared with males by measuring delayed onset of foraging in the presence of indirect chemical cues from a tactile and olfactory hunting vertebrate predator, Elliot’s short-tailed shrew (*Blarina hylophaga*).

Elliot’s short-tailed shrews, typical of the diverse clade of shrews (Eulipotyphla: Soricidae), are small (13–16 g), voracious predators that use rapid speed coupled with a paralyzing, venomous saliva to subdue prey (Thompson et al., 2011). Given their small body size and relatively high mass-specific metabolic rate, shrews require nearly their entire body mass in food each day to avoid starvation (Saarikko, 1989). To consume this high quantity of food, shrews engage in hourly foraging excursions in which arthropods compose the bulk of their diet (Saarikko, 1989; Ritzi, Bartels & Sparks, 2005). Although able to detect chirping crickets, shrews primarily rely on tactile and olfactory senses to locate prey as they patrol established territories (Saarikko, 1989; Platt, 1976; Pernetta, 1977). Due to their increased motile behavior, female crickets are theoretically more likely than males to be preyed upon by shrews. Given shrews are habitat generalists with abundant yet locally patchy distributions (Thompson et al., 2011), there is a potential that unequal predation risk between cricket sexes could alter sex ratios and lead to dynamic shifts in cricket metapopulations. To determine if female crickets recognize and associate a higher predation risk from mobile, non-acoustically hunting insectivores disproportionately to males, we made use of chemical cues produced by the shrew to measure indirect predation risk assessment.

Shrews possess numerous, highly odiferous abdominal glands for marking territories and deterring predators (Thompson et al., 2011). These chemical markings could be used by crickets as an indicator of shrew presence in the wild. Chemical recognition of predators has evolved in a wide variety of predator–prey systems as a mechanism for prey to indirectly assess predation risk (Kats & Dill, 1998). This behavior has previously been observed in field crickets (*Gryllus integer*), with experimental evidence that juveniles can recognize and avoid arachnid predators via chemotactile signals alone (Kortet & Hedrick, 2004). Additionally, adult house crickets (*Acheta domesticus)* have been shown to recognize centipedes via olfaction (Hoefler, Durso & McIntyre, 2012). While these patterns have never been extended to mammalian predators, we hypothesized chemical recognition of predators is likely a behavior that transcends taxonomy. By experimentally manipulating shrew odor within a habitat and measuring anti-predator response behavior, (i.e., delayed foraging) we directly tested if (1) adult crickets detect and respond to shrew odor as an indicator of predation risk and (2) if female crickets, with a hypothesized higher risk towards shrew predators, display more pronounced anti-predator response of delayed foraging than males.

## Materials & Methods

We used commercially bred house crickets (*Acheta domesticus*) purchased from Fluker’s Cricket Farm (Port Allen, LA, USA). This species is native to Southwest Asia, although, it has subsequently spread throughout much of the globe through human-aided dispersal (Ghouri, 1961; Weismann & Rentz, 1977; Alexander, 1957). Within North America, large populations of feral house crickets have been established throughout the southern portion of the continent, including the southern Missouri, Arkansas, and Mississippi River basins, where populations overlap with those of Elliot’s short tailed shrews (Thompson et al., 2011). Purchased crickets were first acclimated for one week within a 50 L communal container, containing approximately 1,000 crickets. The container was filled with ample cover via egg cartons and was free of known predator stimulus. Conditions were maintained at 20 °C with a 12 h light/dark photoperiod, during which the crickets were fed sliced apples and oranges and watered ad libitum. 24 h prior to experimental trials, a subset (*n* = 15–25) of healthy (i.e., large, no missing limbs) adult crickets were randomly selected and moved to a new container where they were deprived of food and water.

Trials were conducted in two opaque, 36 × 16 × 16 cm, sturdy plastic study chambers. Pieces of brown, 0.5 mm thick, corrugated cardboard were cut from a single source sheet to fit into the bottom of the plastic study chamber (36 × 16 cm). Cardboard pieces were maintained within a sterile environment until needed for trials. 24 h prior to conducting trials, one cardboard sheet was infused with shrew odor by association with a captive Elliot’s shrew for 24 h. Cardboard was placed on the bottom substrate of one-half of the shrew enclosure, a 50 gallon aquaria (91 × 46 × 48 cm), so that the paws and ventral surface of the shrew, including abdominal scent glands, would have direct contact with the cardboard. This tactile interaction with the cardboard was observed while the shrew regularly patrolled its’ enclosure. Care was taken to ensure the cardboard was not placed near the shrew’s latrine sites to minimize excretion based chemical signals. The shrew was originally captured from Hays, KS (KDWP&T permit number SC-118-2011) and housed at the Sternberg Museum of Natural History for education. The shrew enclosure was held at a constant 20 °C with a 12 h light/dark photoperiod during the 24 h odor infusion period. In captivity, the shrew’s diet consisted of mealworms, crickets, oats, and peanut butter. Odor infused cardboard was ensured to be free of feces and other particulates and transferred to the chamber with clean forceps. Control cardboard pieces were transferred directly from the sterile environment to the control study chamber with clean forceps. No cover was provided within the study chambers.

At the onset of trials, a slice of fresh orange was placed in one corner of each chamber. One cricket at a time, chosen at random without regard for sex, was released in the chamber, opposite from the orange, and time to observed foraging activity was recorded with a stopwatch. Observations were done live and in-person, with care taken to not disrupt cricket behavior. In the event that a cricket did not forage within 720 s (chosen arbitrarily a priori), the trial was concluded and the individual was treated as censored (Rich et al., 2010). Following confirmed foraging or timed-out censorship, crickets were removed from the chamber along with any debris introduced by the cricket. Cardboard pieces were not replaced between each individual’s trial, instead they were replaced at semi-regular intervals following approximately 15–25 trials. Although it was possible crickets also encountered conspecific chemical cues during trials, crickets were acclimated communally, and such cues would not be considered novel. Additionally, there is evidence that conspecific chemical cues alone are not enough to induce behavioral responses in *Acheta domesticus* (Hardy & Shaw, 1983). Trials were repeated until 100 crickets (50 male and 50 female) were run through each treatment, odor and control, for a total of 200 crickets with no individual being used in more than one trial. Trials were completed over the span of 3 weeks in October of 2011. All trials were completed at 20 °C, under fluorescent lighting between 15:00 and 19:00 h. Although the lighting conditions were neither natural or reflective of when crickets are most active (typically either late afternoon or pre-dusk, Nowosielski & Patton, 1963; Cymborowski & Muszyńska, 1974), these conditions were necessary to aid in visual confirmation of foraging and other behaviors.

We conducted a Kaplan–Meier survivorship analysis to assess if time to foraging (i.e., anti-predator behavior) differed between male and female crickets in both the odor and control chambers. Specifically, we used the Gehan-Breslow-Wilcoxon log-rank method in the survival package of program R (Therneau, 2015; R Core Team, 2016) to test if the proportion of crickets foraging was different between four treatments: males and females within each chamber type, odor, and control. This method is appropriate due to the non-constant rate of delayed foraging and the skew of all censored (i.e., timed-out) individuals taking place at 720 s (Rich et al., 2010). Four pairwise comparisons, between males and females within each chamber type and among males and females between chamber types, were planned a priori and adjusted for multiple comparisons with a Bonferroni correction.

## Results

**Figure 1:**
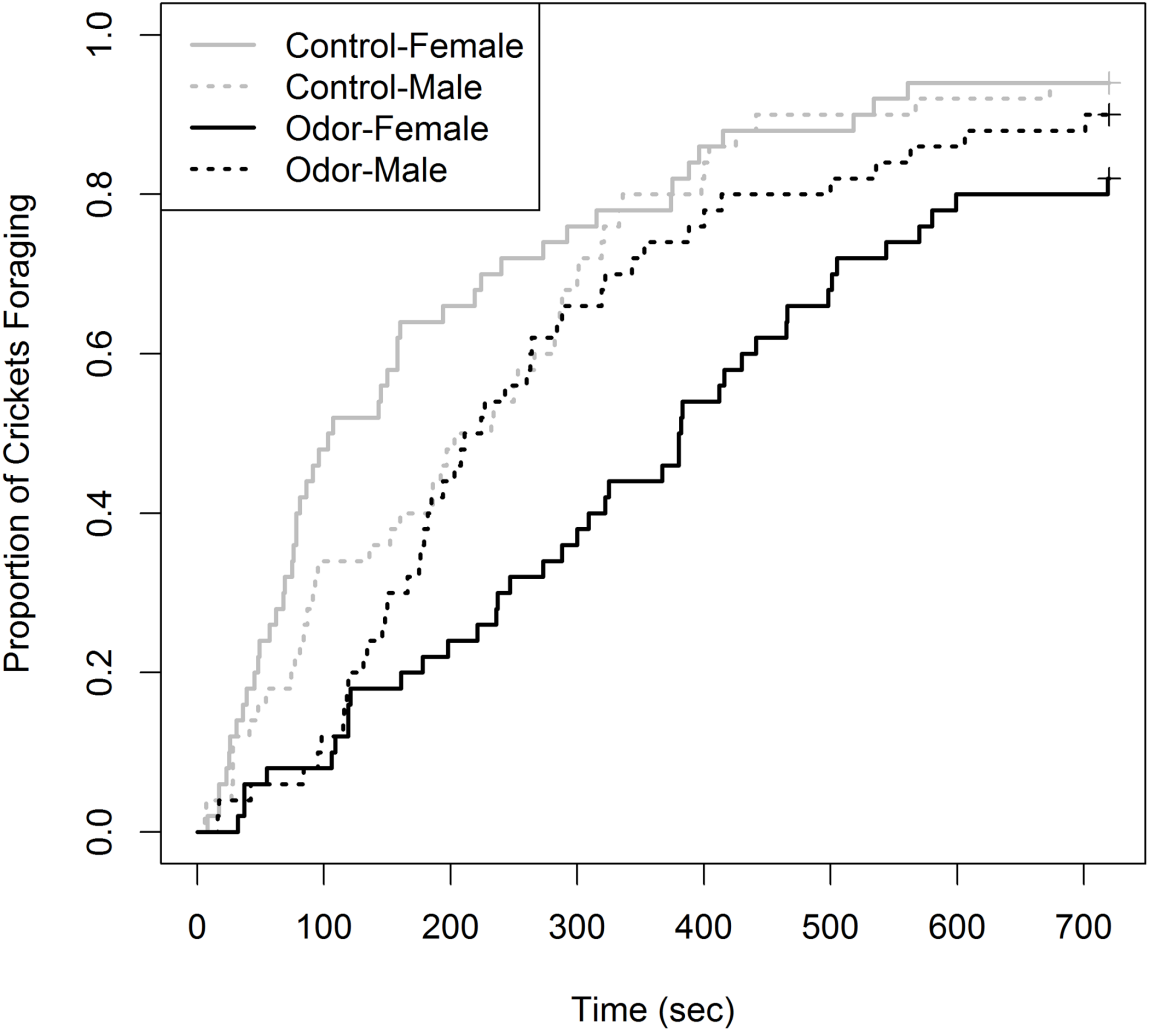
Proportion of house crickets (*Acheta domesticus*) which foraged within the presence of either a control or Elliot’s shrew (*Blarina hylophaga*) odor. The proportion of commercial house crickets (*Acheta dometicus*) which foraged, at the expense of other anti-predator behaviors, in the presence of either a control or Elliot’s shrew (*Blarina hylophaga*) odor. 50 male and 50 female crickets, unique for each treatment (odor and control) were individually exposed to treatment and timed until foraging began. Individuals which did not forage during the 720 sec trial were removed and treated as censored data, indicated by pluses.

Of the 200 total crickets tested, 20 individuals were censored (10% of total sampled). This could have been reduced by increasing the time until censorship to >720 s; however, an adequate sample size was attained for Kaplan–Meier analysis. Censorship was unevenly split, with six occurring in the control group (3 female, 3 male) and 14 in the odor group (nine female, five male). The Kaplan–Meier curves showing the proportion of crickets foraging over time for the four treatments were not identical (*χ*^2^ = 26.4, *df* = 3, *p* < 0.001), with female crickets in the odor and control group occupying the upper and lower extremes, respectively (Fig. 1). Cricket behaviors observed during trials predominantly included continuous movement and antennae touching of the cardboard substrate until feeding. Within the odor chamber, crickets of both sex would often approach food multiple times prior to feeding. Males in both experimental treatments never made acoustic signals. However, it should be noted these observations reflect general trends and were not scored or quantified rigorously.

Of the pairwise comparisons, we detected no difference in the foraging curves between females and males of the control group (*χ*^2^ = 2.6, *df* = 1, *p* = 0.42). There was a non-significant trend for females to have a greater foraging latency than males in the shrew odor treatment (*χ*^2^ = 6.0, *df* = 1, *p* = 0.058). We did not detect a difference between males within the control and odor groups (*χ*^2^ = 1.2, *df* = 1, *p* = 1.00). Finally, there was a significant difference between females within the odor and control groups, where females displayed greater latency of foraging within the shrew odor treatment (*χ*^2^ = 21.2, *df* = 1, *p* =  < 0.001). It should be noted that the power of the Gehan-Breslow-Wilcoxon log-rank method to detect differences between some groups is limited due to the non-constant rate of the proportion foraging within each group, indicated by crossed foraging curves (Fig. 1).

## Discussion

Once detected by predators, crickets’ primary defense strategies involve rapid-burst speed to escape or the voluntary loss of limbs (i.e., autotomization). Given this limited arsenal for predator defense, obtaining advanced and indirect recognition of predation risk is vital for assessing habitats and reducing costly anti-predator behaviors. Our results provide strong evidence that female crickets are able to detect chemical cues that correspond with shrew presence and delay foraging for vigilance accordingly. This contributes to the already lengthy list of prey species that use chemical signals to decrease predation risk, underscoring the importance of chemosensation in predator–prey evolution (Kats & Dill, 1998).

Interestingly, male crickets do not appear to alter foraging rates when in the presence of shrew chemical signals. It is unclear, however, if the lack of response by male crickets indicates the inability to detect the chemical cues or indifference to the signal. This could be elucidated in subsequent studies by adding one or more negative control odors to the experimental design. Not only could this provide evidence for the range of chemical perception with male crickets, but it could also help confirm if responses from females were shrew-odor specific or more general. Few studies have explored chemosensory detection of predators within crickets; unfortunately, previous studies did not test for differences between sexes, either using juveniles of unidentified sex or adults of a single sex (Kortet & Hedrick, 2004; Storm & Lima, 2008; Hoefler, Durso & McIntyre, 2012; Binz et al., 2014; Atwell & Wagner, 2015). Male crickets have been documented to identify and respond to pheromones from females and potentially recognize differences in sympatric species (Otte & Cade, 1976), as well as use chemosensory perception for foraging (Matsumoto & Mizunami, 2000). Regardless of the mechanism, the absence of response to the chemical signals of shrews supports our hypothesis that anti-predator behaviors would be greater within female crickets, as they have higher predation risk from non-acoustically hunting predators.

Although domestic crickets and Elliot’s shrews have experienced overlapping ranges within North America for at least half a century, the evolutionary history of domestic crickets occurred on continental Asia. While this might suggest a recent adaptation to the recognition of shrew odor as a signal for predation risk, it should be noted that the shrew family (Soricidae) has a global distribution (Fumagalli et al., 1999; Dubey et al., 2007) and scent glands are common trait throughout the group (Eadie, 1938; Hutterer, 1985). Furthermore, shrews are not unique in being highly odiferous; numerous mammalian insectivores spanning several distinct taxonomic clades and geographic distributions also possess highly pungent scent glands (Poduschka & Wemmer, 1986; Haffner, 1998; Dawley, Miller & Schnader, 1999; Jannett & Jannett, 2009). Given the near ubiquity of scent-marking among small insectivorous mammals and the evolutionary histories of both domestic crickets and Elliot’s shrews, it is highly likely that our results are not a species-specific interaction but instead a general response of crickets. Previous studies have also suggested that chemosensory recognition of predators is directly related to the individual predator’s consumption of conspecifics (Hoefler, Durso & McIntyre, 2012). Further work using a variety of mammalian and control odors would help differentiate the generality of our results across taxa and predation risk.

Although this is the first study to explicitly test cricket response to a highly mobile, mammalian predator, previous work has been done on cricket boldness using variety of other predation stimuli (Kortet & Hedrick, 2004; Hedrick & Kortet, 2006; Wilson et al., 2010; Hoefler, Durso & McIntyre, 2012; Niemelä, DiRienzo & Hedrick, 2012; Binz et al., 2014). Findings from many of these studies run counter the trends we observed. For instance, past studies reported that latency to emerge from shelter in novel environments and following simulated predation events were consistently higher among male crickets (Hedrick & Kortet, 2006; Hedrick & Kortet, 2012; Niemelä, DiRienzo & Hedrick, 2012). Likewise, Wilson et al. (2010) demonstrated that female crickets are typically more active when exploring novel areas. However, these experiments all occurred within a laboratory setting and were likely free of any predator chemical stimuli. In the absence of chemical stimuli within our study, females appeared to initially forage faster than males (Fig. 1), although the entire foraging curves did not significantly differ. This further highlights the pronounced behavioral changes among female crickets in the presence of a predator’s chemical cues.

The apparent dichotomy between the responses of either sex to different hunting strategies, observed both from our results and prior studies, suggests interesting implications for community dynamics through predator control of cricket sex ratios. Alterations to the operational sex ratio can drastically impact the amount of sexual selection and intraspecific competition within a population (Souroukis & Murray, 1994; Murray & Cade, 1995). This can lead to quantifiable alterations in population genetics and evolutionary trajectories (Bull, 1981; Souroukis & Cade, 1993).

While female crickets significantly delayed foraging in favor of increased vigilance in the presence of shrew odor, we did not test the effectiveness of this as an anti-predator behavior. It should be noted that crickets used in this study were commercially bred and likely naive to shrew odor, meaning that the perception of predation risk and behavioral responses observed are innate rather than learned. While commercial crickets do possess similar behavioral traits to their wild counterparts (Wilson et al., 2010; Fisher et al., 2015), subsequent studies on natural populations would be necessary to firmly establish the relationship suggested here and to determine the ecological significance of our findings. Habitat choice or giving-up density experiments could provide additional value to determining how influential chemical cues from shrews, or other mammalian predators, are to crickets when making decisions about predation risk and the impact this has on broad ecological and evolutionary patterns.

##  Supplemental Information

10.7717/peerj.4923/supp-1 Supplemental Information 1 Time to foraging for crickets in odor and control treatments

## Additional Information and Declarations

### Data Availability

The following information was supplied regarding data availability: The raw data have been supplied as Supplementary File.
